# Exploring the Lived Experiences of Young Women With Congenital Heart Disease Through Adolescence: A Qualitative Feminist Study Using Focus Groups

**DOI:** 10.1111/hex.14179

**Published:** 2024-09-18

**Authors:** Anna Tylek, Charlotte Summers, Ellen Maulder, Lindsay Welch, Lynn Calman

**Affiliations:** ^1^ University Hospital Southampton NHS Foundation Trust, School of Health Sciences University of Southampton Southampton UK; ^2^ Lay Contributor With Lived Experience of Congenital Heart Disease UK; ^3^ Faculty of Health and Social Sciences, Department of Nursing Bournemouth University Bournemouth UK; ^4^ School of Health Sciences, Centre of Psychosocial Research in Cancer University of Southampton Southampton UK

**Keywords:** adolescence, CHD, congential heart disease, feminist, lived experiences, mental health

## Abstract

**Objectives:**

The overarching aim of this study is to explore, examine and identify the experience that young women with congenital heart disease face as they transition through adolescence into womanhood.

**Design:**

This is an empirical qualitative study conducted in the form of three focus groups. The study design and analysis adopted a feminist ontological positioning to elucidate the voice of women and offer an alternative perspective of cardiology health care. Data were analysed using the inductive thematic approach informed by the study aims.

**Participants:**

A group of seven female participants (mean age 26) based in the United Kingdom, each with varying degrees of congenital heart defects that required open heart surgery growing up, was included in the study.

**Results:**

Three key themes with antecedent concepts emerged: (a) the impact of womanhood and the potential influence of motherhood on the young women themselves transitioning through adolescence with CHD within medical and sociocultural contexts, (b) the challenges of being a woman and undergoing heart surgery during adolescence on the young women's health before, during and after surgery and (c) the effect of existing online/offline healthcare and social structures on women's health during transitioning through adolescence These themes were encompassed under an overarching theme of psychological complexities developed throughout the cardiac journey from diagnosis through to post‐surgery.

**Conclusion:**

This study built on the limited exploration of being a young woman and having CHD and confirmed that there are vulnerabilities and challenges in having CHD as a young woman transitioning through adolescence. This was a result of sex (biological characteristics) and gender factors (socially constructed roles). This leads to short‐ and long‐term implications on psychological well‐being. This research indicates that enhancements are needed in the provision of care and psychological support for young women with CHD. This will help to enable women to achieve a good quality of life in addition to increased life expectancy offered by medical advancements.

**Patient or Public Contribution:**

Active participant involvement was crucial to ensure the authentic female voice in the study. This study received support from young women with congenital heart disease. Young women contributed to the study design, recruitment of participants and analysis of results. Two of the women were also co‐authors of this paper.

## 1 Introduction

Congenital heart disease (CHD), affecting approximately 0.8%–1.2% of live births worldwide [1, 2] encompasses various birth defects affecting heart function [3]. Fortunately, advancements in surgery, anaesthesia, diagnostic techniques and ongoing research [4] have improved survival rates, allowing more children to transition into adolescence [1, 5]. However, challenges persist in the quality of life for adolescents with CHD, emphasising the importance of understanding their experiences during the transition to adulthood.

The World Health Organisation (WHO) defines adolescence as the period from 10 to 19 years of age, marked by crucial psychological development, identity formation, moral awareness, shifts in relationships, pursuit of independence and cognitive and emotional maturation [6]. For children with CHD, this stage often brings increased understanding of their diagnosis presenting psychosocial challenges [7, 8, 9, 10, 11, 12, 13, 14]. These challenges include physical issues such as reduced physical functioning [8]; physical symptoms such as chest pain [7]; social issues such as feeling different from peers [9, 13], parental protection [10] and bullying [14]; emotional issues such as loneliness; and coping with uncertainty [8, 11]. Existing research has explored the experiences of both genders concurrently, contributing to a comprehensive understanding of adolescents with CHD [15].

However, feminist literature argues that despite certain similarities, women and men are fundamentally different due to sex (biological characteristics) and gender (societal factors), significantly shaping their lived experiences [16, 17]. These distinctions have been somewhat evident in the existing body of literature on CHD. For example, women have specifically reported issues surrounding fertility [7, 14], contraception [7], body image [7, 8, 9, 13] and concerns regarding relationships [7, 13, 14].

Moreover, research has demonstrated that women with CHD may encounter distinct medical challenges during adolescence. These include delayed pubertal milestones, such as later onset of menarche [18, 19]. Another concern arises from the limited availability of contraception options, as contraceptive methods containing oestrogen are contraindicated for women with cyanotic CHD [20]. Pregnancy‐related complications, including arrhythmias, irreversible heart failure and even mortality, can also be risks [21]. Moreover, the impact of CHD complications extends to offspring, contributing to issues like prematurity, increased foetal mortality risk and a higher likelihood of CHD recurrence in future generations [22]. Additionally, as women with CHD get older, they face a higher prevalence of frailty and cognitive dysfunction, exacerbating the need for comprehensive healthcare approaches tailored to their specific needs [15].

Two studies have examined some of the experiences that young women with CHD may encounter. Nakamura et al. [23] shed light on challenges such as appearance issues and apprehension regarding dating and starting a family, often accompanied by feelings of guilt if their CHD were to impact their child's health. Nakamura et al. [23] observed that cultural influences may shape a young woman's perspective on living with CHD, as they found that the guilt associated with CHD's influence was less prevalent in Western nations compared to Japan. This suggests that young women with CHD in the United Kingdom may approach family planning differently than their counterparts in Japan.

Similarly, Gantt [24] addressed issues, including pregnancy concerns, mortality fears, body image concerns, parental protection and contraception challenges. While offering valuable insights into the experiences of young women, it is essential to acknowledge that healthcare practices and societal expectations have evolved significantly since its publication. Consequently, some of the findings may not directly align with the current landscape, particularly with the rise of social media introducing new beauty standards adversely affecting the self‐esteem of young women [25]. Therefore, when designing healthcare interventions and support systems for young women with CHD today, it is essential to consider these evolving factors.

Although existing literature on adolescents with CHD touches on sex‐ and gender‐specific issues and provides a medical perspective on the complications of CHD in women, there remains a notable gap in research exploring these complications from young women's perspectives. Consequently, their experiences are often generalised and serve to conflate the differing experiences between young men and women. This has implications for the provision of individualised care as the specific needs of young women are not adequately addressed. This emerging evidence underscores the importance of further investigation to comprehensively understand the role of sex‐ and gender‐specific issues in the context of CHD. Bridging this knowledge gap is essential to improving care for young women with CHD. Therefore, we aim to explore the experiences of young women with CHD through a feminist lens to enhance the provision of care and support, ultimately enabling them to achieve a good quality of life in addition to increased life expectancy offered by medical advancements.

## 2 Methods

### 2.1 Ontological Positioning

This study explored young women's experiences of transitioning with CHD through adolescence by using a feminist standpoint epistemology [26] in the study design and as an interpretive approach to data. Despite the absence of a single definition of feminist research, Cook and Fonow [26] outline five epistemological principles. These include the consideration of women and gender as the focus of analysis, the importance of raising awareness, the rejection of subject and object, addressing the power imbalance between the researched and the researcher, prioritising ethics and seeking to empower women [26].

By adopting feminist principles, the experiences of women were placed at the core of the study, with an emphasis on understanding the ways in which their biological body and gender have shaped their experiences of CHD. This allowed for an exploration of an alternative view on the current health care, allowing for specific recommendations for the female population.

### 2.2 Study Design

This empirical qualitative study comprised three focus groups. Although no method is intrinsically feminist, feminist researchers who have utilised focus groups have commended their alignment with feminist principles [27]. This is due to their relatively naturalistic nature [28], allowing for the exploration of lived experiences among women. Moreover, with participants outnumbering the researcher, power dynamics shift towards the participants, empowering them [28, 29] by giving them a ‘voice’ that stimulates more situated discussions.

### 2.3 Sample

**Figure 1:**
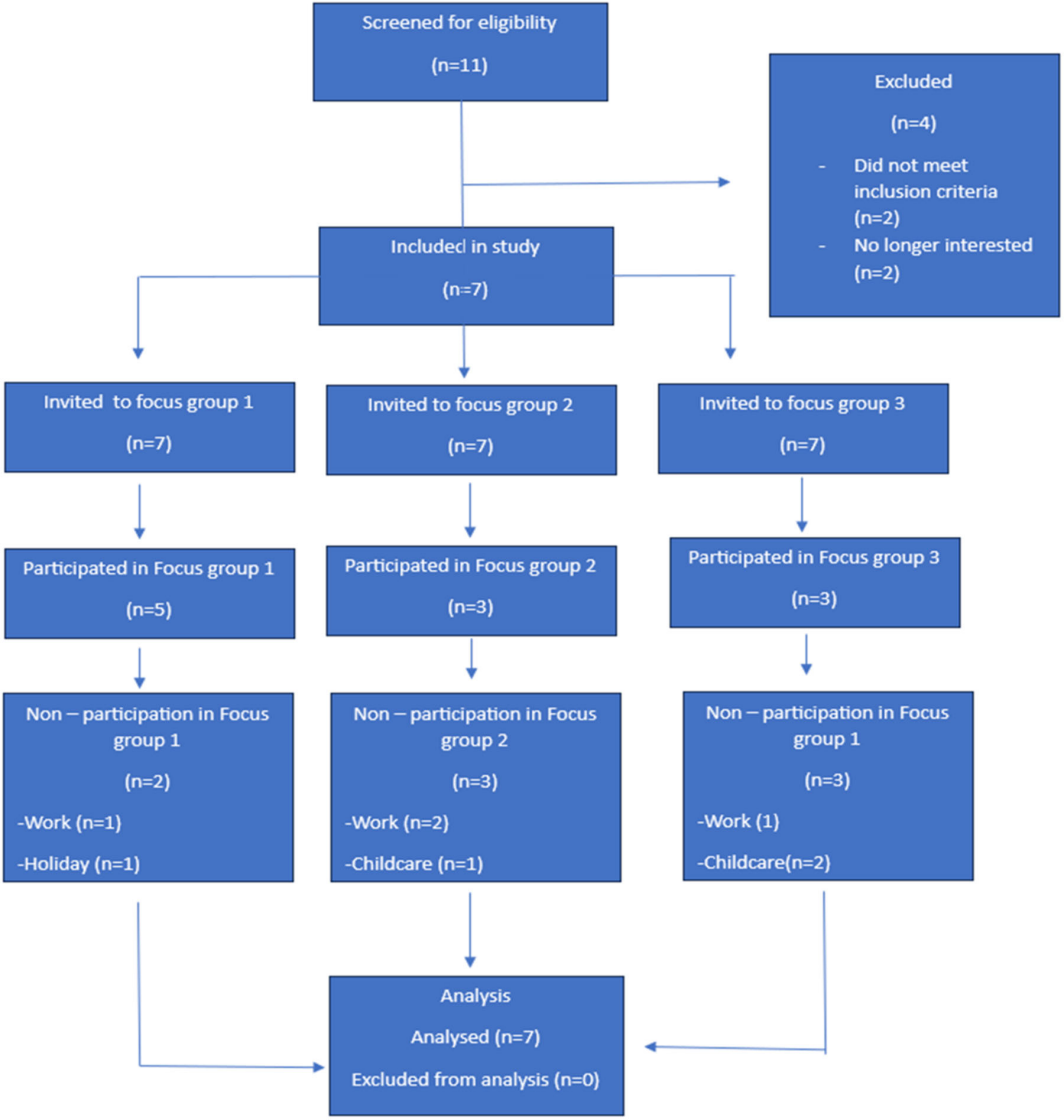
Flowchart highlighting the recruitment process.

Young females with CHD aged 19–30 years, having undergone open‐heart surgery since infancy and throughout childhood, who could converse in English and provide consent, were eligible to participate. We excluded young females who had multiple health conditions other than CHD and who were deemed too unwell to partake based on mental and physical health. A diagram has been developed to demonstrate the recruitment process (Figure 1).

### 2.4 Recruitment

In adherence to the recommended focus group size of six to eight participants, a combination of convenience and snowball sampling methods was used through community connections. The recruitment process received assistance from Hannah Philipps, the author of a published patient story [30]. Initially, Philipps assisted in enlisting the initial cohort of participants, who subsequently aided in the identification of additional participants. All potential participants received a participant information sheet and had the option to electronically sign a consent form if they chose to participate. Participants were informed that this research was part of the researcher's Master's degree programme, and the lead researcher received regular supervision and support from a post‐doctoral supervisor (L.W.).

### 2.5 Ethics

Ethical approval (ERGOII: 66509) was granted by the University of Southampton's Ethics and Research Governance Online (ERGO). Committee Written consent was obtained before the study and reconfirmed on the day of the focus groups, with participants reminded of their right to withdraw.

### 2.6 Data Collection

Between December 2021 and January 2022, three Microsoft Teams focus groups were conducted, led by an undergraduate female student nurse (A.T.) with support from a female research supervisor (L.W.). The discussions centred on transitioning to womanhood, experiences with the current healthcare system and mental health challenges related to CHD. Discussion prompts were used to encourage natural conversations and recall of experience.

Each group included three to seven participants, and nonattendance was often attributed to childcare and work commitments (Figure 1). Sessions lasted approximately 90 min, concluding when no new discussion topics arose, indicating data saturation. Focus groups were audio‐ and video‐recorded and anonymised on transcription, with data retention for several years before permanent removal as per ethical guidelines.

### 2.7 Data Analysis

The recordings were transcribed verbatim. Thematic analysis [31] approached from a feminist standpoint [32, 33] was independently conducted by the researcher following the six phases [31]. Thematic analysis was chosen due to its ability to explore a diverse range of experiences among participants, thus helping to yield themes [31].

An inductive approach was adopted, as it tends not to be driven by the preconceived theoretical interest as demonstrated by the deductive approach but is data‐driven, hence prioritising the voice of young women [31, 34]. Transcripts were read several times before initial coding, allowing for familiarisation with the data. Initial coding was developed on a line‐by‐line basis utilising the traditional pen and paper method. Codes were then grouped into potential themes to ensure that they accurately reflected their lived experiences, enhancing the trustworthiness and credibility of the findings.

The data were examined through a feminist lens drawing from feminist scholars [16, 28] to investigate how gender and the biological body confine a woman's lived experiences. It is important to note that although a feminist lens guided the analysis, the respondents never questioned the patriarchal views and therefore experienced health care in a male‐dominated world.

### 2.8 Methodological Rigour

The researcher acknowledged that their position as a student nurse and personal experiences can influence the research. Therefore, reflexivity was practised to ensure validity and quality by recognising biases and examining the implications of those biases. Regular meetings with the supervisor and participants were held to discuss ongoing analysis and reach a consensus on developing themes. The Standards for Reporting Qualitative Research (SRQR) checklist was followed [35].

### 2.9 Patient and Public Involvement

Active participant involvement was crucial to ensure the authentic female voice in the study. This study received support from young women with congenital heart disease. Young women contributed to the study design, recruitment of participants and analysis of results. Two of the women were also co‐authors of this paper.

## 3 Results

**Figure 2:**
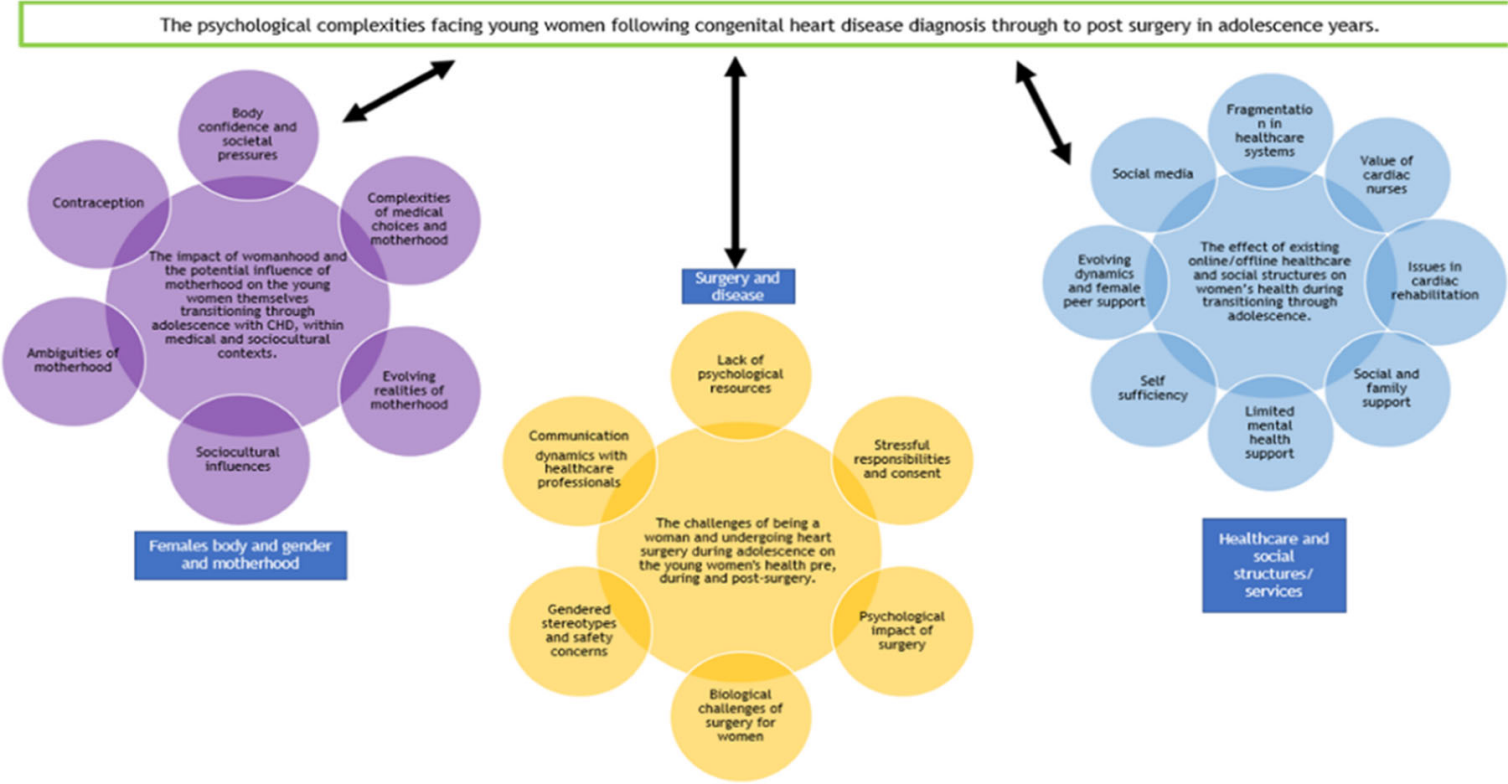
Visual representation of the themes and the overarching themes.

Three key themes with antecedent concepts emerged from the analysis that captured the experiences of seven young women with CHD transitioning through adolescence. Figure 2 provides a visual representation of the themes. These themes were encompassed under an overarching theme of psychological complexities developed throughout the cardiac journey from diagnosis through to post‐surgery, which is woven throughout the quotations of respondents in the three themes. To structure the reporting of respondents' experiences, results are presented utilising categories determined during the analysis. Although themes are discussed individually, their interrelated nature and direct impact on each other are emphasised.

Participants' direct quotations are presented in italics, with participants' details being presented in a pseudonymised form. Ellipses indicate where inapplicable data have been omitted. See Table 1 for an overview of the themes and subthemes.

**Table 1:** Summary of themes and subthemes.

Theme	Subthemes
The impact of womanhood and the potential influence of motherhood on the young women themselves transitioning through adolescence with CHD, within medical and sociocultural contexts.	– Sociocultural influences. – Body confidence and societal pressures. – Complexities of medical choices and motherhood. – Contraception. – Ambiguities of motherhood. – Evolving realities of motherhood.
The challenges of being a woman and undergoing heart surgery during adolescence on the young women's health before, during and after surgery.	– Lack of psychological resources. – Stressful responsibilities and consent. – Psychological impact of surgery. – Biological challenges of surgery for women. – Gendered stereotypes and safety concerns. – Communication dynamics with healthcare professionals.
The effect of existing online/offline healthcare and social structures on women's health during transitioning through adolescence.	– Fragmentation in healthcare systems. – Value of cardiac nurses. – Issues in cardiac rehabilitation. – Social and family support. – Limited mental health support. – Self‐sufficiency. – Evolving dynamics and female peer support. – Social media.

### 3.1 The Female Body and Gender and Motherhood

The impact of being a female is analysed in terms of gender (societal expectations of a female) and sex (biological characteristics) [16].

#### 3.1.1 Sociocultural Influences

Young women expressed the challenges of navigating societal expectations where women are increasingly expected to excel in both professional careers and domestic responsibilities. For women with CHD, these dual expectations often necessitate difficult choices about which role to prioritise. This leads to women negotiating their role and responsibilities, taking into account both their health constraint and societal expectations regarding their gender role.

Working full time, having children and trying to run a house is not sustainable and that is not something that I'll be able to do, so it's a compromise of looking at what is going to suffer.

P06

#### 3.1.2 Body Confidence and Societal Pressure

Societal expectations influenced young women's self‐confidence, leading to practices like wearing high‐neck tops or undergoing surgical procedures to conform to the ‘idealised’ female body.

About the age of 15‐16, I was really low and just really self‐conscious, so I ended up having plastic surgery on it to make it a bit neater.

P02

Several of the older participants expressed acceptance in their journey with their bodies; however, this was frequently challenged by societal views that harmed their self‐confidence.

I'm quite happy I get my chest out, I'm not fussed … but I had a family member comment and I've had some negative experiences with men before commenting like you should just cover up like who wants to see that sort of thing and that made me more determined to be like f*** you. But I think it really affected me.

P06

#### 3.1.3 Complexities of Medical Choices and Motherhood

In a medical setting, respondents reported the battle between the available medical care options versus being a female with CHD. Motherhood, a societal expectation of women, often served as a limiting factor for young females and healthcare professionals while making medical decisions.

There is vulnerability as a young woman in having this disease … different types of surgeries don't equate well to young women. So, if I wanted to have a mechanical valve transplant, I then increase my risk of miscarriage by X per cent … I have a friend who is about to go through her fourth operation because she tried everything and she has to just compromise and be like if I wanna live a normal life, I've got to go on warfarin which highly decreases her chances of ever having children.

P01

### 3.2 Contraception

This was further affirmed by the availability of contraception to young women with CHD.

Not being able to take X contraception because you're on this beta‐blocker or not being able to do that because you could get a blood clot. Like you are just limited. I had to have the coil fitted the other day. Did I want to? No. Was it the only thing that I had left? Yes. So, it's happening.

P01

Contrary to health contradictions such as blood clots, most respondents reported being put on the wrong type of contraceptive.

I was put on the wrong type for many years. I've gotten the combination pill and it's only later that I was told that I should be on progesterone‐only because of the high risk of clots and stuff so that was not great.

P02

### 3.3 Ambiguities of Motherhood

Motherhood and becoming a mother, a personal and often natural experience for women, seemed extremely medicalised and controlled, with elements of fear and concern about the potential complications, surgeries and changing expectations.

Really scares me. Uh, just the prospect that likes it might not happen for me. Or I might not be able to. Or it could do wrong or even just like if I were to get pregnant, I might have to have surgery again before … It's a bit of a concern like it does worry me, it does upset me.

P04

I've always been told I need to speak to my cardiac team when I'm thinking of trying.

P04

### 3.4 Evolving Realities of Motherhood

Furthermore, medical choices concerning motherhood were often contradicting and ever‐changing, causing an added emotional burden on the young females, as the reality of motherhood that they had come to terms with was frequently revised.

I grew up believing that and when I was 24, I went for the first time to my appointment with my partner and they were like ‘oh when are you planning your pregnancy then?’ And I was told I couldn't have children naturally and they were like ‘oh yeah you can’ and I just like this is major, what? ‘Cause I had it set in my head that I'm gonna adopt, it's gonna be fine, actually it's probably better that I don't put my body through though this. I felt overwhelmed’.

P06

## 4 Disease and Surgery

### 4.1 Psychological Complexities Surrounding Surgery

This section reveals the psychological complexities emerging before, during and after surgery, highlighting the difficulties that females encounter in the surgical process.

### 4.2 Lack of Psychological Preparation

Respondents reported feeling medically ready but socially and psychologically unprepared for surgery during adolescence, emphasising the impact on their formative years.

When we have that timing of the operation, like that medical intervention, we leave it to a point that we're fully grown to do the intervention but it's just really a bad time to do it because they are your founding years, the years you learn the most. They are the years your body is gonna be affected mentally.

P01

### 4.3 Stressful Responsibilities and Consent

The stress surrounding surgery increased with the responsibility of signing consent forms and being accountable for one's own treatment, a task that respondents found emotionally challenging.

I had to sign my consent form and that was horrific. Like the day before meeting my surgeon … My parents couldn't interfere, they were there but, they couldn't say anything because I was the one signing and I remember him being like on the risks and the top word was like death … and that taking off that kind of responsibility, I could have just done without it.

P01

### 4.4 Psychological Impact of Surgery

Respondents' recollection of their mental health before, during and after surgery revealed dissociation, existential reflections on mortality and a sense of depression.

I think I was just in shock, and it was obviously a lot … like three to six months on the lead up to the operation I was just so empty. I had no feelings whatsoever … I felt like my body was walking around and I wasn't there … I felt very detached from myself and my surroundings for quite a while.

P01

I have this input on my mortality that nobody else gets like my friends and family like they just don't have that same perspective like you literally sit there and look it in the eye, and I don't know anybody else who's done that … it changes you and that is what causes that level of depression.

P04

### 4.5 Lack of Psychological Support

Although respondents experienced psychological complications, they reported that their health was solely driven by their physical state and no psychological support was provided.

There's not really any support beyond physical support afterwards and that's what's really hard.

P04

## 5 Biological Challenges of Surgery for Females

Additional difficulties with surgery were noticed in the biological component of being a female. Results revealed that the physical manifestation of becoming a woman was perpetually at odds with CHD.

### 5.1 Impact on Body Image and Breasts

Respondents detailed the physical and emotional challenges of surgery on breasts, including pain exacerbated by the weight and the desire for cosmetic interventions.

It was horrendous, like the weight of my boobs made the pain so much worse. And like lying in bed and it just felt like it was pulling constantly.

P04

… I was going to have a boob job because I wanted to make my boobs even again.

P01

### 5.2 Menstrual Cycle Disruption

Surgery‐induced stress disrupted the young females' menstrual cycle, further complicated by psychological vulnerabilities and the challenge of receiving care from male healthcare professionals.

A male nurse woke me up ‘cause there was just blood all over the bed where my period had started because of the stress.

P04

### 5.3 Gendered Stereotypes and Safety Concerns

Additionally, as young females approached adolescence, gendered stereotypes such as men being depicted as dangerous [36] began to bear heavily on them. Respondents faced psychosocial vulnerabilities such as feeling unsafe around male figures when they were in a vulnerable state, this being doctors and patients, and often requested female healthcare professionals (HCPs) to provide personal care.

I had to request to get put into a private room because it was really like overwhelming and like just men around.

P01

I was quite traumatised by the fact that like a male doctor has undressed me when I was unconscious.

P01

### 5.4 Communication Dynamics With Healthcare Professionals

Although communicating with female healthcare professionals was perceived as easier due to shared anatomy and experiences, having male doctors did not pose a significant barrier in discussing care‐related matters.

I don't remember it being particularly uncomfortable. I do think there's maybe a lack of understanding as a man of the impact on you as a woman … like confidence and scars and stuff I never would have felt like I could bring that up with a male doctor because they just won't get it. But yeah, not a huge issue having male doctors.

P04

## 6 Healthcare and Social Structures/Services

### 6.1 Healthcare System Challenges

This section explores the problems with the current CHD healthcare service.

### 6.2 Fragmentation in Healthcare Systems

There was general agreement that the present healthcare infrastructure was often challenging to navigate due to fragmentation between services and the multidisciplinary team. Lengthy communication processes between services were noted, emphasising the significance of a liaison cardiac nurse in addressing this issue.

I had to like go through the wringer and phone the department. They put me in contact with this person. They then sent a letter to my GP; my GP went back to my cardiologist, and I was like this could be easily solved if I had a cardiac nurse.

P01

### 6.3 Value of Cardiac Nurses

Respondents who had access to cardiac nurses noted the nurses' value in integrating health care. Several respondents reported the simplicity of contact and the ability to gain support directly without contacting multiple services.

They're really easy to get a hold of, so you've got a direct line or bleep, and they always get back to you, … At the start of COVID, I wanted to check if I need to do anything different, so I just emailed them, and they got to me really quickly.

P03

## 7 Issues in Cardiac Rehabilitation

### 7.1 Inappropriateness for Young Females

In terms of the services within the current infrastructure such as cardiac rehabilitation, which provides exercise training and health education guidance, all respondents believed that it was not appropriate for young adolescents, as they were in a room with individuals much older who had differing life, social and personal circumstances.

You're surrounded by people who are like 50 plus that have had a heart attack, and it's a completely different ball game mentally and physically.

P06

Respondents experienced discrimination from the older attendees which prevented them from taking full advantage of the programme since they felt uncomfortable and alienated.

I went twice, and I never went back because I was made to feel so uncomfortable as a young person with a heart disease.

P01

### 7.2 Lack of Mental Health Focus

Furthermore, respondents felt that the cardiac rehabilitation programme was solely focused on physical health, whereas ‘mental health rehab is what we need’ P04.

### 7.3 Mental Health Support and Self‐Sufficiency

This section discusses the lack of mental health support within the healthcare system, leading young females to be self‐sufficient in seeking psychological support.

### 7.4 Limited Mental Health Support

Lack of mental health support was echoed throughout the cardiac journey from diagnosis through to post‐surgery. Respondents reported that the psychological aspect of living with CHD was often neglected, and they received no support.

But definitely, mental health. There was nothing, like at all. I didn't even get a leaflet.

P01

### 7.5 Self‐Sufficient in Seeking Psychological Support

Due to the lack of established mental health support, young females have had to be self‐sufficient in gaining psychological support. Some respondents accessed private counselling but owing to financial constraints, this was not sustainable.

I did 4 sessions but then I couldn't afford any more.

P04

## 8 Social and Family Support

This section explores the significant role of social and family support, particularly the influence of maternal figures and the evolving dynamics as young females mature.

### 8.1 Maternal Support

A lot of support has therefore been through social support. Family, particularly maternal figures, plays a significant part in providing emotional and social support to young females, contributing significantly to their strength and perspectives of their condition.

She was always like ‘you're fine, come on, you're right, you can do it, you can do what they can do’ … I owe a massive amount of my strength and my views on my condition and everything else to my mum for sure.

P04

### 8.2 Evolving Dynamics and Peer Support

As young females matured, they reported wanting to protect their family from the reality of their feelings by trying to look brave.

I'd tried to hold everything together for my family because it was bad enough … they didn't need to see how bad it had affected me on top of the physical stuff … I just tried to kind of hide that from them.

P04

This led to increased reliance on peer support through social media platforms like Instagram and Facebook groups.

There's a lot of support groups and stuff on Facebook I use at the moment and different pages. I would say that does help.

P07

### 8.3 Female Network

In particular, female networks of support were important, as they allowed respondents to connect with other young females who had comparable surgeries, and thus offer emotional and social support to one another.

… best thing ever because it meant that I didn't feel alone and those silly questions that you don't feel like you can ask your parents or your doctor about I could go to her.

P01

In addition, young women reported that they benefitted from the focus group and from sharing their experiences with other young women.

So actually thank you for you as well because I think without it, I wouldn't have realized those things so.

P01

It's really reassuring to in in a really weird way to know that people have had the same experiences as you, and you're not, you're not dramatizing it in your head.

P06

### 8.4 Caution With Social Media

Although social media was appreciated and viewed as a source of positive support, some respondents stressed the importance of being cautious when using it.

… you have to be careful ‘cause I think sometimes they can be really negative, or people put false information in there.

P04

## 9 Discussion

This is the first empirical feminist research to explore the experiences of young women with CHD transitioning through adolescence. Furthermore, the study design and analysis adopted a feminist ontological positioning to elucidate the voice of women and offer an alternative perspective of cardiology health care [16], which is dominated by the male voice, with 86 per cent of consultants being male [37]. This study built on the limited exploration of being a woman and having CHD and confirmed that there are vulnerabilities and challenges in having CHD as a woman, which disempowers young women in today's society. This was due to sex (biological characteristics) and gender factors (societal influences) [16].

Our results build on the existing literature on the challenges faced by young women with CHD, including body image concerns [7, 8, 9, 13], contraceptive access difficulties [7, 24] and pregnancy concerns [23, 24]. Nevertheless, through a feminist lens, we contribute new findings by exploring how having a female body and being a woman impact the experience of CHD.

The female transitioning period into adolescence is influenced by sex. Young women experience physical changes such as breast development and the onset of menarche, posing additional challenges during this phase [38]. However, for young women with CHD, these challenges are compounded, leading to complications like irregular menstrual cycles, limited contraceptive choices [18, 19] and heightened discomfort due to breast development impacting surgical scars. These findings highlight how CHD attacks the female body and deprives young women of freedom, such as the opportunity to choose from various contraceptive choices, which are associated with women's empowerment as they enhance their decision‐making authority [39].

Furthermore, our findings explored how the socially constructed role of women exacerbates social, clinical and medical challenges resulting in gender‐related disadvantages. Society's expectations for women include motherhood, employment and adhering to certain physical standards [40]. However, for women with CHD, these ideals are often unattainable due to CHD conflicting with societal expectations of a woman. This resonates with findings from Moola, Norman [41], who reported that the impact of CHD and cystic fibrosis on the valued social roles was a concern for young women. Our study illustrates how young women struggle with accepting their scars due to societal beauty standards and face discrimination for not fitting the conventional ‘female image’.

Further, they faced psychosocial vulnerabilities such as feeling unsafe in adult wards when surrounded by male patients and receiving personal care from male healthcare providers. Prior research supports these vulnerabilities, with Lindsay et al. [42] highlighting vulnerability among adolescents with acquired brain injury and Groutz et al. [43] exploring women's preferences during breast examination.

Additionally, Morton et al. [44] observed that hospital gowns induced feelings of vulnerability, disempowerment and self‐consciousness in females compared to their male counterparts. These findings demonstrate that young women are faced with psychological complexities throughout their CHD journey due to CHD being at odds with their body and their position in society.

Our findings highlight the complexity of transitioning through adolescence as a young woman with CHD in a medical and social setting. Considering the limited support available, respondents found comfort in female figures, such as maternal figures during childhood and peers during adulthood. This echoes the findings of Abrams et al. [45], who identified mothers as a key source of emotional and social support. Respondents also valued peer support, enabling them to share experiences and navigate the challenges inherent in being a young woman with CHD. This corresponds with previous research highlighting the beneficial role of peer support in managing medical conditions [46, 47].

## 10 Strengths and Limitations

The use of a feminist lens in this study significantly enriched the understanding of the lived experiences of young women with CHD by emphasising gendered experiences, promoting empowerment and incorporating intersectionality. Despite potential limitations, such as the risk of bias and overemphasis on gender, the study managed to balance these by exploring comprehensive factors such as mental health concerns.

The study had a relatively small sample size, with data collection ending upon recognising data saturation, indicated by the absence of new discussion. It remains uncertain whether additional focus groups involving different participants would have yielded additional topics for discussion. However, Malterud et al. [48] and Yardley [49] argue that the richness of the findings and the information power are more important than the size of the sample. Thus, the small sample allowed participants to feel safe when discussing their lived experiences, as reflected in their candid and vulnerable discussions.

Many of the women interviewed were self‐referred, providing valuable insights from individuals who actively sought to share their experiences. This proactive participation highlights the importance and relevance of the study to those directly affected. Although this may limit the generalisability of the findings to all women with CHD, it ensures that the experiences gathered are deeply engaged and highly relevant. Furthermore, it provides a strong starting point for addressing these issues on a wider scale in future research. This enabled us to reach a seldom heard group of individuals, gain rich data and engage them in research that has provided outcomes that benefit the female health and healthcare system.

## 11 Implications for Practice

Presently, health care has yet to implement systematic changes that prioritise gender‐ and sex‐specific interventions. However, emerging research has underlined the importance of considering and integrating sex, gender and their interactions into healthcare policies [50, 51, 52]. A recent initiative by the UK government [53] has acknowledged the underrepresentation of women in research and commits to a comprehensive review, placing women at the forefront of healthcare considerations.

By elucidating the female voice, our findings establish the need for a care model that acknowledges sex and gender as integral components of the individual's CHD experience. This will contribute to the quality of life of young women in addition to the increased lifespan that medical advancements offer.

HCPs should provide female patients with practical and psychosocial support. Care plans should be developed that address female‐specific issues and offer psychological support. Currently, no psychological support is embedded into the care pathway for CHD as observed in diabetes [54] and cancer [55]. Introducing psychological support can serve as a positive coping strategy for young women transitioning through adolescence and should be embedded throughout the CHD journey. Table 2 provides further recommendations for different areas of care.

**Table 2:** Recommendations for healthcare professionals.

Area of care	Recommendations
Psychological support	– Integrate psychological support into the CHD care pathway. – Provide access to mental health professionals who understand the unique challenges faced by young women with CHD. – Offer group therapy or support groups specifically for young women with CHD.
Gender‐sensitive care	– Encourage the presence of female healthcare providers when requested by patients to enhance comfort and trust. – Train healthcare professionals to understand the gender‐specific impacts of CHD, including societal expectations and gender roles.
Body image and self‐esteem	– Address body image concerns proactively, offering resources such as counselling or support groups. – Acknowledge and validate the patient's feelings about societal standards and the impact on self‐confidence.
Social support networks	– Encourage involvement of family in the care to provide emotional and social support. – Facilitate connections with peer support networks.
Education	– Educate young women on self‐advocacy and their condition. – Ensure ongoing education for healthcare professionals on the needs of women with CHD.
Safe and inclusive environment	– Create a safe and inclusive environment by ensuring privacy and comfort. – Request promptly to requests for gender‐specific accommodations.
Healthcare infrastructure	– Simplify navigation of the healthcare system by providing liaison cardiac nurse to coordinate care. – Adjust cardiac rehabilitation programmes to be age‐appropriate and inclusive for young women.
Motherhood and contraception	– Provide comprehensive counselling on contraceptive options and their implication for women with CHD. – Offer individualised plans that address the concerns related to pregnancy and motherhood, considering both medical and psychological impacts.

## 12 Conclusion

In conclusion, our findings highlight the abundance of challenges and obstacles that young women with CHD confront, emphasising the unique framework of difficulties that they face due to CHD and surgery survival. These women often endure psychological and physical difficulties due to receiving male‐oriented clinical care that neglects their specific needs. It is crucial to address this gap by implementing strategies to understand and improve health outcomes for women with CHD. This includes offering practical recommendations for clinicians to enhance their care and interactions with female adolescents and women living with congenital heart disease.

## Author Contributions

Anna Tylek devised, conducted and analysed the research, with Dr Lindsay Welch as her primary research supervisor. Lindsay Welch, reviewed and contributed to the writing of the ethics, conduct of the study, analysis and write up. Lynn Calman reviewed the publication and supported the manuscript. Charlotte Summers and Ellen Mauler contributed as public contributors with lived experienced, and contributed to reviewing the analysis.

## Conflicts of Interest

The authors declare no conflicts of interest.

## Data Availability

The data that support the findings of this study are available from the corresponding author upon reasonable request.

## References

[1] “National Congenital Heart Disease Audit,” National Institute of Cardiovascular Outcomes Research (NICOR), (2023), https://www.nicor.org.uk/wp-content/uploads/2022/06/NICOR-NCHDA_2022-FINAL.pdf.

[2] W. Wu , J. He , and X. Shao , “Incidence and Mortality Trend of Congenital Heart Disease at the Global, Regional, and National Level', 1990–2017,” Medicine 99, no. 23 (2020): e20593, https://www.ncbi.nlm.nih.gov/pmc/articles/PMC7306355/. 32502030 10.1097/MD.0000000000020593 PMC7306355

[3] “Congenital Heart Disease,” National Health Service (NHS), (2018), https://www.nhs.uk/conditions/congenital-heart-disease/.

[4] S. L. Kang and L. Benson , “Recent Advances in Cardiac Catheterization for Congenital Heart Disease,” *F1000Research* 7 (2018): 370, https://www.ncbi.nlm.nih.gov/pmc/articles/PMC5871969/pdf/f1000research-7-14119.pdf. 10.12688/f1000research.13021.1 PMC5871969 29636905

[5] “Children and Young People Statistics 2013,” British Heart Foundation (BHF), (2013), https://www.bhf.org.uk/informationsupport/publications/statistics/children-and-young-people-statistics-2013.

[6] J. M. Sales and C. E. Irwin Jr., “A Biopsychosocial Perspective of Adolescent Health and Disease,” in Handbook of Adolescent Health Psychology, eds. W. T. O'Donohue , L. T. Benuto , and L. W. Tolle (New York, NY: Springer New York, 2013), 13–29.

[7] L. S. H. Chong , D. A. Fitzgerald , J. C. Craig , et al., “Children's Experiences of Congenital Heart Disease: A Systematic Review of Qualitative Studies,” European Journal of Pediatrics 177, no. 3 (2018): 319–336, https://link.springer.com/article/10.1007/s00431-017-3081-y. 29327140 10.1007/s00431-017-3081-y

[8] M. G. Pagé , A. H. Kovacs , and J. Irvine , “How Do Psychosocial Challenges Associated With Living With Congenital Heart Disease Translate Into Treatment Interests and Preferences? A Qualitative Approach,” Psychology & Health 27, no. 11 (2012): 1260–1270, https://www.tandfonline.com/doi/pdf/10.1080/08870446.2012.667099?casa_token=sa_Dt7Osp38AAAAA:R0kjPsd-eRdL6PDaVbiamjJGMsAzXzan-FlrGwOIL-Xz2L3mNFZYCVl6czV3Oj1VwGy8kPHzWKIj. 22433017 10.1080/08870446.2012.667099

[9] K. Shearer , G. R. Rempel , C. M. Norris , and J. Magill‐Evans , “ ‘It's No Big Deal’: Adolescents With Congenital Heart Disease,” Journal of Pediatric Nursing 28, no. 1 (2013): 28–36, https://www.sciencedirect.com/science/article/pii/S0882596312001297?casa_token=SIsP85SAo60AAAAA:APOrJlh_X-EBEctCIfVKOOHz02TH2Tn2qWiViQ5G5vESMlH9lL3gGEQ. 22543260 10.1016/j.pedn.2012.03.031

[10] E. M. Tong , P. S. A. Sparacino , D. K. H. Messias , D. Foote , C. A. Chesla , and C. L. Gilliss , “Growing Up With Congenital Heart Disease: The Dilemmas of Adolescents and Young Adults,” Cardiology in the Young 8, no. 3 (1998): 303–309, https://www.cambridge.org/core/journals/cardiology-in-the-young/article/abs/growing-up-with-congenital-heart-disease-the-dilemmas-of-adolescents-and-young-adults/891F4B9CCF49C75D53933166ED09CB4E. 9731644 10.1017/s1047951100006806

[11] S. K. Tye , G. Kandavello , S. A. W. A. Badwi , and H. S. A. Majid , “Challenges for Adolescents With Congenital Heart Defects/Chronic Rheumatic Heart Disease and What They Need: Perspectives From Patients, Parents and Health Care Providers at the Institut Jantung Negara (National Heart Institute) Malaysia,” Frontiers in Psychology 11 (2021): 1–9, https://www.frontiersin.org/journals/psychology/articles/10.3389/fpsyg.2020.481176/full. 10.3389/fpsyg.2020.481176 PMC7873049 33584393

[12] M. Berghammer , M. Dellborg , and I. Ekman , “Young Adults Experiences of Living With Congenital Heart Disease,” International Journal of Cardiology 110, no. 3 (2006): 340–347, https://www.sciencedirect.com/science/article/pii/S016752730501048X. 16226816 10.1016/j.ijcard.2005.08.006

[13] Y. Birks , P. Sloper , R. Lewin , and J. Parsons , “Exploring Health‐Related Experiences of Children and Young People With Congenital Heart Disease,” Health Expectations 10, no. 1 (2007): 16–29, https://onlinelibrary.wiley.com/doi/epdf/10.1111/j.1369-7625.2006.00412.x. 17324192 10.1111/j.1369-7625.2006.00412.x PMC5060379

[14] Y. T. Chiang , C. W. Chen , W. J. Su , et al., “Between Invisible Defects and Visible Impact: The Life Experiences of Adolescents and Young Adults With Congenital Heart Disease,” Journal of Advanced Nursing 71, no. 3 (2015): 599–608, https://onlinelibrary.wiley.com/doi/epdf/10.1111/jan.12546. 25296699 10.1111/jan.12546

[15] B. Daelman , L. Van Bulck , K. Luyckx , et al., “Frailty and Cognitive Function in Middle‐Aged and Older Adults With Congenital Heart Disease,” Journal of the American College of Cardiology 83, no. 12 (2024): 1149–1159, https://www.jacc.org/doi/10.1016/j.jacc.2024.01.021. 38508848 10.1016/j.jacc.2024.01.021

[16] K. Aranda , Feminist Theories and Concepts in Healthcare: An Introduction for Qualitative Research (Bloomsbury, London: Macmillan International Higher Education, 2017).

[17] A. M. Barry and C. Yuill , Understanding the Sociology of Health: An Introduction (London: Sage, 2011).

[18] M. Leroy‐Melamed , A. Katz , and M. L. Shew , “Menstrual Dysfunction and Treatment Among Adolescents With Congenital Heart Disease,” Journal of Pediatric and Adolescent Gynecology 33, no. 6 (2020): 686–690, https://www.sciencedirect.com/science/article/pii/S1083318820303089. 32827759 10.1016/j.jpag.2020.08.012 PMC9134926

[19] M. Vigl , M. Kaemmerer , E. Niggemeyer , et al., “Sexuality and Reproductive Health in Women With Congenital Heart Disease,” American Journal of Cardiology 105, no. 4 (2010): 538–541, https://www.sciencedirect.com/science/article/pii/S000291490902534X. 20152251 10.1016/j.amjcard.2009.10.025

[20] V. Regitz‐Zagrosek , J. W. Roos‐Hesselink , J. Bauersachs , et al., “2018 ESC Guidelines for the Management of Cardiovascular Diseases During Pregnancy,” Kardiologia Polska 77, no. 3 (2019): 245–326, https://academic.oup.com/eurheartj/article/39/34/3165/5078465. 30912108 10.5603/KP.2019.0049

[21] M. Greutmann and P. G. Pieper , “Pregnancy in Women With Congenital Heart Disease,” European Heart Journal 36, no. 37 (2015): 2491–2499, https://academic.oup.com/eurheartj/article/36/37/2491/2466005?login=true. 26112887 10.1093/eurheartj/ehv288

[22] I. M. Van Hagen and J. W. Roos‐Hesselink , “Pregnancy in Congenital Heart Disease: Risk Prediction and Counselling,” Heart 106, no. 23 (2020): 1853–1861, https://heart.bmj.com/content/106/23/1853.abstract. 32611675 10.1136/heartjnl-2019-314702 PMC7677481

[23] M. Nakamura , S. Kita , R. Kikuchi , et al., “A Qualitative Assessment of Adolescent Girls' Perception of Living With Congenital Heart Disease: Focusing on Future Pregnancies and Childbirth,” Journal of Pediatric Nursing 38 (2018): e12–e18, https://www.sciencedirect.com/science/article/pii/S0882596317300088. 29153935 10.1016/j.pedn.2017.11.003

[24] L. T. Gantt , “Growing Up Heartsick: The Experiences of Young Women With Congenital Heart Disease,” Health Care for Women International 13, no. 3 (1992): 241–248, https://www.tandfonline.com/doi/abs/10.1080/07399339209515999. 1399864 10.1080/07399339209515999

[25] Y. Kelly , A. Zilanawala , C. Booker , and A. Sacker , “Social Media Use and Adolescent Mental Health: Findings From the UK Millennium Cohort Study,” EClinicalMedicine 6 (2018): 59–68, https://www.sciencedirect.com/science/article/pii/S2589537018300609. 31193561 10.1016/j.eclinm.2018.12.005 PMC6537508

[26] J. A. Cook and M. M. Fonow , “Knowledge and Women's Interests: Issues of Epistemology and Methodology in Feminist Sociological Research,” Sociological Inquiry 56, no. 1 (1986): 2–29, https://onlinelibrary.wiley.com/doi/abs/10.1111/j.1475-682X.1986.tb00073.x.

[27] S. Wilkinson , “Focus Groups in Feminist Research: Power, Interaction, and the Co‐Construction of Meaning,” in Women's Studies International Forum, vol. 21 (USA: Elsevier Science, 1998), 111–125.

[28] S. Wilkinson , “Focus Groups: A Feminist Method,” Psychology of Women Quarterly 23, no. 2 (1999): 221–244, https://onlinelibrary.wiley.com/doi/abs/10.1111/j.1471-6402.1999.tb00355.x.

[29] B. Pini , “Focus Groups, Feminist Research and Farm Women: Opportunities for Empowerment in Rural Social Research,” Journal of Rural Studies 18, no. 3 (2002): 339–351, https://www.sciencedirect.com/science/article/pii/S0743016702000074.

[30] H. Phillips , “Overcoming Fears in a Life With Congenital Heart Disease,” British Journal of Cardiac Nursing 14, no. 8 (2019): 1–3, https://www.magonlinelibrary.com/doi/abs/10.12968/bjca.2019.0072?journalCode=bjca.

[31] V. Braun and V. Clarke , “Using Thematic Analysis in Psychology,” Qualitative Research in Psychology 3, no. 2 (2006): 77–101, https://www.tandfonline.com/doi/abs/10.1191/1478088706QP063OA.

[32] A. Brooks , “Feminist Standpoint Epistemology: Building Knowledge and Empowerment Through Women's Lived Experience,” Feminist Research Practice: A Primer 1 (2007): 53–82, https://methods.sagepub.com/book/feminist-research-practice/n3.xml.

[33] B. Parry , “Feminist Research Principles and Practices,” in *Online Readings in Research Methods*, eds. S. Kramer , S. Laher , A. Fynn , and H. H. Janse van Vuuren (Johannesburg: Psychological Society of South Africa, 2020), 10.17605/OSF.IO/BNPFS.

[34] M. S. Linneberg and S. Korsgaard , “Coding Qualitative Data: A Synthesis Guiding the Novice,” Qualitative Research Journal 19, no. 3 (2019): 259–270, 10.1108/QRJ-12-2018-0012.

[35] B. C. O'Brien , I. B. Harris , T. J. Beckman , D. A. Reed , and D. A. Cook , “Standards for Reporting Qualitative Research: A Synthesis of Recommendations,” Academic Medicine 89, no. 9 (2014): 1245–1251, https://journals.lww.com/academicmedicine/fulltext/2014/09000/standards_for_reporting_qualitative_research__a.21.aspx. 24979285 10.1097/ACM.0000000000000388

[36] E. A. Bates , K. R. Klement , L. K. Kaye , and C. R. Pennington , “The Impact of Gendered Stereotypes on Perceptions of Violence: A Commentary,” Sex Roles 81, no. 1 (2019): 34–43, https://link.springer.com/article/10.1007/s11199-019-01029-9.

[37] “Census of Consultant Physicians and Higher Specialty Trainees in the UK 2016‐17,” Royal College of Physicians, 2017, https://www.rcplondon.ac.uk/projects/outputs/2016-17-census-uk-consultants-and-higher-specialty-trainees.

[38] K. Schuiling and F. Likis , Gynecologic Health Care, 4th ed. (Massuchsetts USA: Jones & Bartlett Learning, 2020).

[39] A. Alano and L. Hanson , “Women's Perception About Contraceptive Use Benefits Towards Empowerment: A Phenomenological Study in Southern Ethiopia,” PLoS One 13, no. 9 (2018): e0203432, https://journals.plos.org/plosone/article?id=10.1371/journal.pone.0203432. 30212500 10.1371/journal.pone.0203432 PMC6136733

[40] N. Tyagi , R. S. Jha , A. Chaudhary , and S. Batar , “‘Women in Dual Role; a Sociological Perspective,” Ilkogretim Online ‐ Elementary Education Online 20, no. 1 (2021): 1766–1772, 10.17051/ilkonline.2021.01.187.

[41] F. J. Moola and M. E. Norman , “ ‘Down the Rabbit Hole’: Enhancing the Transition Process for Youth With Cystic Fibrosis and Congenital Heart Disease by Re‐Imagining the Future and Time,” Child: Care, Health, and Development 37, no. 6 (2011): 841–851, https://onlinelibrary.wiley.com/doi/abs/10.1111/j.1365-2214.2011.01317.x. 22007984 10.1111/j.1365-2214.2011.01317.x

[42] S. Lindsay , M. Proulx , J. Maxwell , et al., “Gender and Transition From Pediatric to Adult Health Care Among Youth With Acquired Brain Injury: Experiences in a Transition Model,” Archives of Physical Medicine and Rehabilitation 97, no. 2 (2016): S33–S39, https://pubmed.ncbi.nlm.nih.gov/25660004/. 25660004 10.1016/j.apmr.2014.04.032

[43] A. Groutz , H. Amir , R. Caspi , E. Sharon , Y. A. Levy , and M. Shimonov , “Do Women Prefer a Female Breast Surgeon,” Israel Journal of Health Policy Research 5, no. 1 (2016): 35, https://ijhpr.biomedcentral.com/articles/10.1186/s13584-016-0094-3. 27980717 10.1186/s13584-016-0094-3 PMC5131538

[44] L. Morton , N. Cogan , S. Kornfält , Z. Porter , and E. Georgiadis , “Baring All: The Impact of the Hospital Gown on Patient Well‐Being,” British Journal of Health Psychology 25, no. 3 (2020): 452–473, https://bpspsychub.onlinelibrary.wiley.com/doi/full/10.1111/bjhp.12416. 32314508 10.1111/bjhp.12416

[45] A. N. Abrams , E. P. Hazen , and R. T. Penson , “Psychosocial Issues in Adolescents With Cancer,” Cancer Treatment Reviews 33, no. 7 (2006): 622–630, https://www.sciencedirect.com/science/article/pii/S0305737207000126. 10.1016/j.ctrv.2006.12.006 17434265

[46] C. A. Percy , T. Gibbs , L. Potter , and S. Boardman , “Nurse‐Led Peer Support Group: Experiences of Women With Polycystic Ovary Syndrome,” Journal of Advanced Nursing 65, no. 10 (2009): 2046–2055, https://onlinelibrary.wiley.com/doi/abs/10.1111/j.1365-2648.2009.05061.x. 19686401 10.1111/j.1365-2648.2009.05061.x

[47] M. Sallinen , M. L. Kukkurainen , and L. Peltokallio , “Finally Heard, Believed and Accepted–Peer Support in the Narratives of Women With Fibromyalgia,” Patient Education and Counseling 85, no. 2 (2011): e126–e130, https://www.sciencedirect.com/science/article/pii/S0738399111001212. 21419588 10.1016/j.pec.2011.02.011

[48] K. Malterud , V. D. Siersma , and A. D. Guassora , “Sample Size in Qualitative Interview Studies: Guided by Information Power,” Qualitative Health Research 26, no. 13 (2016): 1753–1760, https://journals.sagepub.com/doi/full/10.1177/1049732315617444. 26613970 10.1177/1049732315617444

[49] L. Yardley , “Dilemmas in Qualitative Health Research,” Psychology & Health 15, no. 2 (2000): 215–228, https://www.tandfonline.com/doi/abs/10.1080/08870440008400302.

[50] D. Bartz , T. Chitnis , U. B. Kaiser , et al., “Clinical Advances in Sex‐and Gender‐Informed Medicine to Improve the Health of All: A Review,” JAMA Internal Medicine 180, no. 4 (2020): 574–583, https://jamanetwork.com/journals/jamainternalmedicine/article-abstract/2760346. 32040165 10.1001/jamainternmed.2019.7194

[51] A. Shai , S. Koffler , and Y. Hashiloni‐Dolev , “Feminism, Gender Medicine and Beyond: A Feminist Analysis of ‘Gender Medicine’,” International Journal for Equity in Health 20, no. 1 (2021): 1–11, https://link.springer.com/article/10.1186/s12939-021-01511-5. 34344374 10.1186/s12939-021-01511-5 PMC8330093

[52] S. Sutantri , F. Cuthill , and A. Holloway , “ ‘A Bridge to Normal’: A Qualitative Study of Indonesian Women's Attendance in a Phase Two Cardiac Rehabilitation Programme,” European Journal of Cardiovascular Nursing 18, no. 8 (2019): 744–752, https://academic.oup.com/eurjcn/article/18/8/744/5925397?login=true. 31328533 10.1177/1474515119864208

[53] “Women's Health Strategy: Call for Evidence,” Department of Health and Social Care (UK), 2021, https://www.gov.uk/government/consultations/womens-health-strategy-call-for-evidence/womens-health-strategy-call-for-evidence.

[54] “Diabetes in Children and Young People,” National Institute for Health and Care Excellence (NICE), 2016, https://www.nice.org.uk/guidance/qs125/chapter/Quality-statement-6-Access-to-mental-health-professionals-with-an-understanding-of-type-1-or-type-2-diabetes.

[55] “Cancer Services for Children and Young People,” National Institute for Health and Care Excellence (NICE), 2014, https://www.nice.org.uk/guidance/qs55/chapter/Quality-statement-4-Psychological-and-social-support.

